# PARK7 Protects Against Chronic Kidney Injury and Renal Fibrosis by Inducing SOD2 to Reduce Oxidative Stress

**DOI:** 10.3389/fimmu.2021.690697

**Published:** 2021-05-21

**Authors:** Lijun Yin, Honglin Li, Zhiwen Liu, Wenwen Wu, Juan Cai, Chengyuan Tang, Zheng Dong

**Affiliations:** ^1^ Department of Nephrology, Hunan Key Laboratory of Kidney Disease and Blood Purification, The Second Xiangya Hospital at Central South University, Changsha, China; ^2^ Department of Cellular Biology and Anatomy, Medical College of Georgia at Augusta University and Charlie Norwood VA Medical Center, Augusta, GA, United States

**Keywords:** renal fibrosis, tubular cells, PARK7, reactive oxygen species, antioxidant

## Abstract

Renal fibrosis is the final common pathway to chronic kidney diseases regardless of etiology. Parkinson disease protein 7 (PARK7) is a multifunctional protein involved in various cellular processes, but its pathophysiological role in kidneys remain largely unknown. Here, we have determined the role of PARK7 in renal fibrosis and have further elucidated the underlying mechanisms by using the *in vivo* mouse model of unilateral ureteric obstruction (UUO) and the *in vitro* model of transforming growth factor-b (TGFB1) treatment of cultured kidney proximal tubular cells. PARK7 decreased markedly in atrophic kidney tubules in UUO mice, and *Park7* deficiency aggravated UUO-induced renal fibrosis, tubular cell apoptosis, ROS production and inflammation. In *vitro*, TGFB1 treatment induced fibrotic changes in renal tubular cells, which was accompanied by alterations of PARK7. *Park7* knockdown exacerbated TGFB1-induced fibrotic changes, cell apoptosis and ROS production, whereas *Park7* overexpression or treatment with ND-13 (a PARK7-derived peptide) attenuated these TGFB1-induced changes. Mechanistically, PARK7 translocated into the nucleus of renal tubular cells following TGFB1 treatment or UUO, where it induced the expression of SOD2, an antioxidant enzyme. Taken together, these results indicate that PARK7 protects against chronic kidney injury and renal fibrosis by inducing SOD2 to reduce oxidative stress in tubular cells.

## Introduction

Renal tubulointerstitial fibrosis (TIF) characterized by tubular atrophy, deposition of extracellular matrix and myofibroblast expansion represents the final common pathway to chronic or progressive kidney diseases (CKD) regardless of etiology (1–3). The pathogenesis of renal TIF involves major types of resident renal cells including tubular epithelial cells (TECs), endothelial cells, fibroblasts and pericytes, and also involves inflammatory cells. Accumulating evidence suggests that injury of renal TECs plays a driving role in renal TIF^2,^ (4). After kidney injury, survival TECs undergo proliferation and then redifferentiation to replace damaged or dead TECs to repair renal tubules. Under conditions of severe or repeated injury, renal TECs under phenotypic changes to synthesize and secrete varieties of bioactive molecules that drive interstitial inflammation and fibrosis (2). Thus, understanding the molecular mechanisms responsible for TEC injury is a key to the understanding of the pathogenic mechanism of renal TIF.

Experimental studies have demonstrated that epithelial-mesenchymal transition, cell-cycle arrest at G2/M check points, mitochondrial damage, autophagy, endoplasmic reticulum(ER) stress, and excessive reactive oxygen species (ROS) production contribute to renal tubular cell injury during renal TIF (1–8). Among them, overproduction of ROS appears to be an important pathological factor for renal TIF, which is supported by the findings of ROS accumulation in renal tubules and the beneficial effects of supplementation of exogenous antioxidants in renal TIF regardless of initial etiology (9). Excessive ROS causes oxidative damage to cellular components and organelles including mitochondria, which may lead to cell death. Besides the acute destructive effects of high levels of ROS, a moderate increase of ROS might regulate various signaling pathways that are involved in kidney injury and maladaptive kidney repair, such as the hypoxia-inducible factor 1α pathway, the NLRP3-inflammasome pathway, and the transforming growth factor-β (TGFB1/TGFβ) pathway (10). Investigation of the molecules involved in oxidative stress and the anti-oxidative response in renal tubular cells during renal TIF may provide novel therapeutic targets.

Parkinson disease protein 7 (PARK7/DJ-1) was initially identified as an activated ras-dependent oncoprotein (11), but later studies have implicated PARK7 in the pathogenesis of Parkinson`s disease, diabetes, and male infertility et al (12, 13). Evidence has recently emerged that PARK7 also plays a key role in immune and inflammatory disorders, such as sepsis, and atherosclerosis (14). As a multifunctional protein, PARK7 can act as an antioxidant, transcriptional co-activator, molecular chaperone, deglycase, and/or regulator of signaling pathways (15). PARK7 is abundant in kidneys, particularly in renal tubules (16). Emerging evidence suggests a role of PARK7 in the development of both AKI and CKD (17–20). However, the role of PARK7 in renal TIF and its underlying mechanism remain largely unclear.

In the present study, by using a unilateral ureteric obstruction (UUO)-induced mouse model of renal fibrosis, and culturing renal TECs exposed to TGFB1, we demonstrated that PARK7 protects against renal TIF and related kidney injury by inducing SOD2 and scavenging ROS.

## Materials and Methods

### Antibodies and Reagents

The primary antibodies used in this study include anti-Fibronectin (ab19245) and anti-PARK7/DJ 1(ab18257) from Abcam, anti-cleaved caspase3 (9664), anti-α/β-Tubulin(2148) and anti-GAPDH (5174) from Cell Signaling Technology, anti-Lamin B1 (66095-1-Ig) and anti-Myc tag (6003-2-Ig) from Proteintech, anti-Collagen I (AF7001) from Affinity, anti-α-SMA(A5228) from Sigma-Aldrich, anti-SOD2(sc-137254) from Santa Cruz, and anti‐F4/80 (GB11027) from Servicebio. All secondary antibodies were purchased from Thermo‐Fisher Scientific. Special reagents were from the following sources: CM-H2DCFDA(C6827), Mito-SOX (M36008) and DHE (D11347) from Thermo Fisher Scientific, TUNEL assay kit (12156792910) from Roche Life Science, and recombinant human TGFB1 (GF111) from EMD Millipore. The PARK7-based peptide ND-13 (YGRKKRRKGAEEMETVIPVD) was synthesized by China Peptides (China).

### Cells, Plasmids, Short Interfering RNA, and Transfection

The Boston University mouse proximal tubular cell line (BUMPT) was initially from Drs. William Lieberthal and John Shwartz at Boston University and maintained as previously described (21). BUMPT cells stably expressing *Park7* shRNA were generated by infecting cells with lentivirus encoding *Park7* shRNA followed by selection with puromycin. The shRNA target sequence of *Park7* was 5′-ATCTGGGTGCACAGAATTTAT-3′. The *Park7* shRNA was synthesized by Gene Pharma (Shanghai, China). The PARK7 overexpression plasmid was generated by inserting the coding sequence for C-terminal myc-tagged human *PARK7* into NheI and KpnI enzyme sites of into pcDNA3.1 (+) expression vector. Transfection was performed by using Lipofectamine 2000 reagents (Invitrogen, Carlsbad, MA, USA) under the manufacturer’s instruction. TGFB1 treatment was performed as previously described (22). Briefly, cells were planted in 35mm cell culture dishes to reach >40% confluence by next day. After starving for 12 hours in serum-free high glucose DMEM medium, cells were cultured in DMEM medium containing 0.2% fetal bovine serum and 5 ng/ml of TGFB1. Cells cultured in medium containing 0.2% fetal bovine serum without TGFB1 were as controls. ND13 was dissolved in normal saline and administered at the same time with TGFB1 treatment.

### Mouse Model of Unilateral Ureteral Obstruction

Animal studies were conducted under a protocol approved by the Animal Ethics Committee of the Second Xiangya Hospital of Central South University. Male C57BL/6 mice (8 ~10 weeks old) were from Hunan Slack King Experimental Animal Company (Changsha, China). *Park7* ^tm1Shn^/*Park7* knockout mice were purchased from the Jackson laboratory. Ureteral obstruction (UUO) surgery was performed as described in our recent studies (23). Briefly, mice were anaesthetized with pentobarbital (60 mg/kg) followed by a midline abdominal incision. The left ureter was exposed and separated, and then ligated by using 4/0 silk thread at two different sites in the middle of the ureter. Sham control mice underwent left ureter exposure, separation, but no ligations. For treatment, ND-13 (2 mg/kg of body weight) was administered *via* tail vein injection starting from the day after surgery, daily till the day before euthanasia.

### Histological Analysis of Kidney Tissues

Hematoxylin and eosin (H&E) and Masson’s trichrome staining in kidney sections were performed as previously described (24). Renal tubules with the following histopathological changes were considered as tubular atrophy: tubular epithelial thinning, pyknotic nuclei or tubular dilation, expansion of the interstitial space, with or without protein casts. Tubular atrophy was examined in a blinded manner and scored by the percentage of atrophic tubules: 0, no damage; 1, < 25%; 2, 25-50%; 3, 50-75%; 4, > 75%. For quantification, at least 10 randomly selected fields per mouse and four mice for each group were scored. Aniline blue staining was conducted to label collagen fibrils in kidney tissues. The ratio of positively (blue) stained area to the entire area (excluding the area of glomerular, small vena cava and blood vessels) was quantitated with Image Pro Plus software. At least 10 randomly selected areas with a magnification of 200 x were assessed.

### Immunohistochemical Analysis

Deparaffinized kidney sections were sequentially incubated with 0.1 M sodium citrate for antigen retrieval, 3% H2O2 to block endogenous peroxidase activity and 5% normal goat serum in phosphate-buffered saline to reduce nonspecific binding. The kidney sections were them exposed to a specific primary antibody at 4°C overnight and then biotinylated goat anti-rabbit secondary antibody (Zhongshan Jinqiao Biotechnology, Beijing, China) for 2 hours at room temperature. The signals were detected with a DAB kit. Positive staining was observed by phase contrast microscopy. For quantification, the number of positively stained tubules or cells per mm2 in at least 10 randomly selected fields from each tissue section were evaluated.

### TUNEL Assay

TUNEL assay in kidney tissues and cultured cells was performed as previously described (25). Kidney tissue sections were sequentially deparaffinized, permeabilized, and incubated with TUNEL reaction mixture for 1 h at 37°C in a humidified and dark cassette. Hoechst 33342 staining was conducted to label nucleus. For quantification, the number of TUNEL-positive tubular cells per mm2 in at least 10 randomly selected fields from each tissue section were counted. For TUNEL assay in cultured cells, cells were fixed, and then sequentially subjected to permeabilization, incubation with TUNEL reaction mixture and then Hoechst 33342. The percentage of TUNEL-positive cells in 10 randomly selected fields containing around 200 cells was quantified.

### Isolation of Nuclear Proteins

Isolation of nuclear proteins in cells was conducted by using a kit from Beyotime (China). Briefly, cells were centrifuged at 1000g for 5 minutes, and cell pellets were resuspended in buffer A (10 mM HEPES pH 7.9, 1.5 mM MgCl2, 0.2 mM KCl 0.2 mM phenylmethylsulphonyl fluoride, 0.5 mM dithiothreitol). After incubation on ice for 15 minutes, the suspension was centrifuged at 14,000g for 5 minutes at 4°C to collect the nuclear pellets. The nuclear pellets were resuspended in cold buffer B(20 mmol/l N-2-hydroxyethylpiperazine-N-2-ethanesulfonic acid potassium hydroxide at pH 7.9, 420 mmol/l NaCl, 1.5 mmol/l MgCl2, 0.5 mmol/l dithiothreitol, 25% glycerol, 0.2 mmol/l dithiothreitol) and vortexed every 20 seconds for 30minutes. Finally, nuclear extracts were cleared by centrifugation at 14000g for 10 minutes to collect supernatants for immunoblotting.

### Determination of Reactive Oxygen Species

Mitochondrial ROS and intracellular ROS in BUMPT cells were determined by using Mito-SOX Red (Thermo Fisher Scientific, M36008) and by CM-DCFDA (Invitrogen, C6827), respectively. ROS in kidney tissues was measured by dihydroethidium (Thermo Fisher Scientific, D11347) staining as previously described (26). Briefly, after harvesting, kidneys were snap frozen by liquid nitrogen. 20 mm sections of the kidneys were incubated with 10 mM DHE in a humidified and dark chamber at 37°C for 30 min and then counterstained with DAPI (Sigma-Aldrich, D9542). Images of the above staining were captured with fluorescence microscopy. For quantification, the fluorescence intensity in the nuclei of proximal tubular cells within 10 random optical sections was determined with ImageJ software. At least 10 randomly selected fields per mouse and four mice for each group were examined.

### Immunofluorescence Staining

BUMPT cells were collected and washed once with phosphate-buffered saline (PBS), followed by fixation with 4% paraformaldehyde. After additional washing with PBS, fixed cells were sequentially underwent permeabilization with 0.1% Triton X-100, blocking with 5% bovine serum albumin hydration, and incubation with primary antibodies overnight at 4°C. And then cells were exposed to Alexa-conjugated secondary antibodies(Abcam, Cambridge, MA). Eventually, the sample were examined under fluorescence microscopy.

### Immunoblotting Analysis and Real-Time PCR

Cells or renal cortical tissues were lysed with 2% SDS buffer (63 mM Tris-HCl, 10% glycerol, and 2% SDS) containing protease inhibitor cocktail (Sigma-Aldrich, P8340). Immunoblot analysis was conducted as previously described (27). RNA extraction and quantitative real-time PCR analysis were described in our recent studies (28). The sequences for the primers used in this study are listed below:

*Park7* forward, 5′-AGTCGCCTATGGTGAAGGAGATCC-3′,
*Park7* reverse, 5′-TGAGCCAACAGAGCCGTAGGAC-3′,
*Tgfβ* forward, 5′-GAGCCCGAAGCGGACTACTA-3′,
*Tgfβ* reverse, 5′-GTTGTTGCGGTCCACCATT-3′,
*Tnfα* forward, 5′-CAGGCGGTGCCTATGTCTC-3′,
*Tnfα* reverse, 5′-CGATCACCCCGAAGTTCAGTAG-3′,
*Sod2* forward, 5′- AAGGGAGATGTTACAACTCAGG-3′,
*Sod2* reverse, 5′- GCTCAGGTTTGTCCAGAAAATG-3′,
*Gapdh* forward, 5′- AGGTCGGTGTGAACGGATTTG -3′,
*Gapdh* reverse, 5′- GGGGTCGTTGATGGCAACA -3′.

### Statistics

Statistical analysis was conducted by using GraphPad Prism software. Multiple comparisons with ANOVA and student’s t-test were applied to determine statistical differences in multiple groups and two groups, respectively. Quantitative data in the present study are representatives of at least 3 independent experiments and are expressed as means ± SEM. P < 0.05 indicates significant differences.

## Results

### PARK7 Is Reduced in Renal Proximal Tubules Following UUO

**Figure 1:**
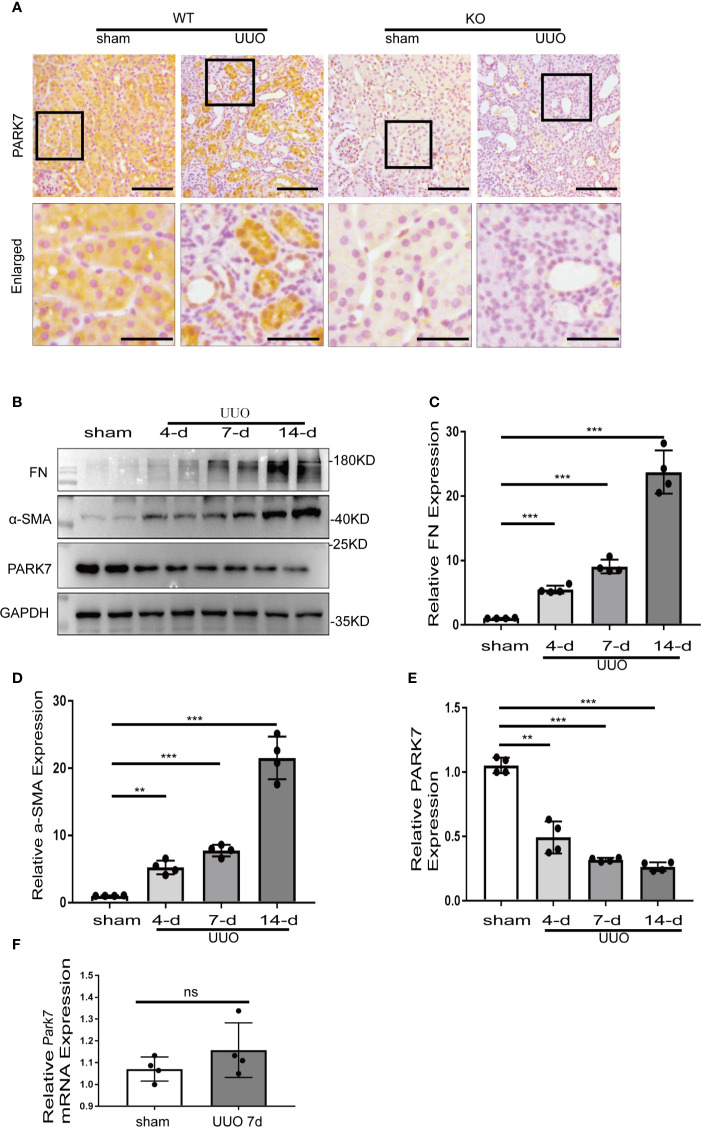
PARK7 is reduced in renal proximal tubules during UUO. C57BL/6 mice (female, 8weeks old) were subjected to sham operation or UUO surgery. The mice were euthanized at 4 days, 7 days or 14 days after surgery, and the UUO-obstructed kidneys were collected. **(A)** Representative images of histochemical staining of PARK7 in kidney tissues. Bar: 100μm in the top images and 50μm in the bottom enlarged images. **(B)** Representative blots of Fibronectin (FN), α-SMA, PARK7 and GAPDH(as protein loading control). **(C–E)** Densitometry of FN, α-SMA, and PARK7 signals(n=4). The signal of FN, α-SMA, or PARK7 was normalized to the GAPDH signal of the same samples to determine the ratios. The values are expressed as fold change over sham control. Each symbol (circle) represents an individual mouse. **(F)** Quantification of Park7 mRNA levels. Error bars: SEM. **p < 0.01, ***p < 0.001. ns, not significant.

We first evaluated the levels of PARK7 during renal TIF in a mouse model of unilateral ureteral obstruction (UUO). Immunohistochemical analysis of kidney sections showed that in sham-operation mice, PARK7 was abundant in renal tubules, and mainly localizes in cell cytosol (**Figure 1A**). Compared to sham-operation mice, UUO mice had high levels of PARK7 in intact renal tubules, but atrophic tubules with dilation showed an obvious decrease of PARK7 (**Figure 1A**). In addition, the intact renal tubules appeared to have more PARK7 in cell nucleus (**Figure 1A**). Immunoblot analysis showed that UUO induced renal expression of fibrosis marker proteins like fibronectin and alpha-smooth muscle actin (α-SMA) in a time-dependent manner, while the expression of PARK7 was decreased (**Figures 1B–E**). Furthermore, We investigated the mRNA level of *Park7* and found that UUO did not alter its transcriptional process (**Figure 1F**). These results indicate an overall reduction of renal PARK7 during renal TIF.

### UUO-Induced Renal Interstitial Fibrosis Is Aggravated in *Park7-*Knockout Mice

**Figure 2:**
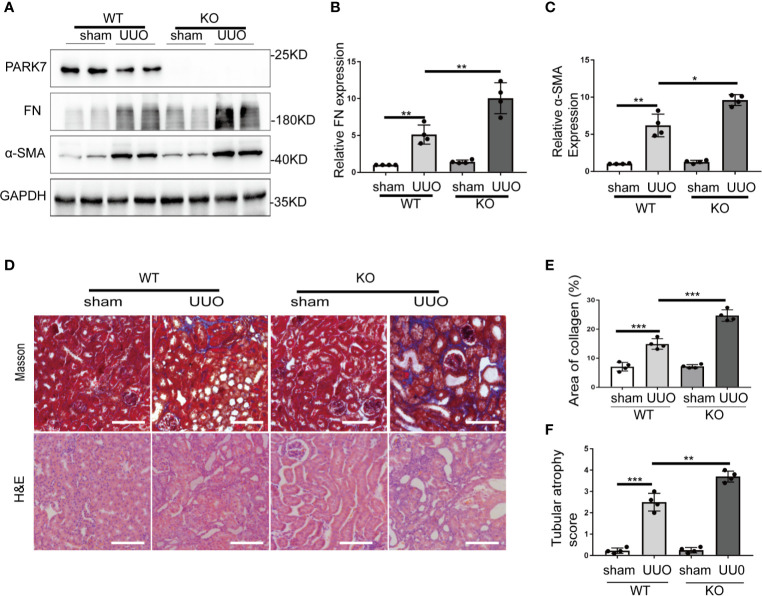
PARK7 deficiency aggravates UUO-induced renal interstitial fibrosis. *Park7* knockout (KO) and wildtype (WT) mice were subjected to either sham operation or UUO surgery. The mice were euthanized at 7 days and UUO-obstructed kidneys were collected. **(A)** Representative blots of PARK7, Fibronectin (FN), α-SMA and GAPDH(as a loading control). **(B, C)** Densitometry of FN and α-SMA signals(n=4). The signal of FN, α-SMA was normalized to the GAPDH signal of the same samples to determine the ratios. The values are expressed as fold change over sham control in WT mice. **(D)** Representative images of Masson’s trichrome staining and hematoxylin and eosin (H&E) staining. Bar: 100μm. **(E)** Quantitative analysis of Masson’s trichrome staining. **(F)** Tubular atrophy score(n=4). Tubular atrophy was graded by 0, 1 (1%‐25%), 2 (26%‐50%), 3 (51%‐75%), 4 (76%‐100% tubules showing atrophy. Each symbol (circle) represents an individual mouse. Error bars: SEM. *p < 0.05, **p < 0.01, ***p < 0.001.

To determine the role of PARK7 in renal TIF, *Park7* knockout (KO) mice were applied. Day 7 after UUO surgery, the obstructed kidneys from wildtype (WT) or *Park7* ko mice were collected. Immunoblot analysis demonstrated that *Park7* ko mice had higher renal levels of fibronectin and α-SMA than WT mice after UUO (**Figures 2A–C**). Masson’s trichrome staining was performed to detect collagen deposition, and the results revealed that *Park7* ko mice had an increase of renal collagen deposition compared to WT mice after UUO (**Figures 2D, E**). In addition, quantification of tubular atrophy in kidney sections stained with hematoxylin and eosin (H&E) demonstrated that following UUO, had tubular atrophy was more severe in *Park7* ko mice than in WT mice (**Figures 2D, F**). Collectively, these findings suggest that *Park7* deficiency aggravates UUO-induced renal TIF.

### *Park7* Deficiency Increases Tubular Cell Apoptosis and Inflammation In UUO

**Figure 3:**
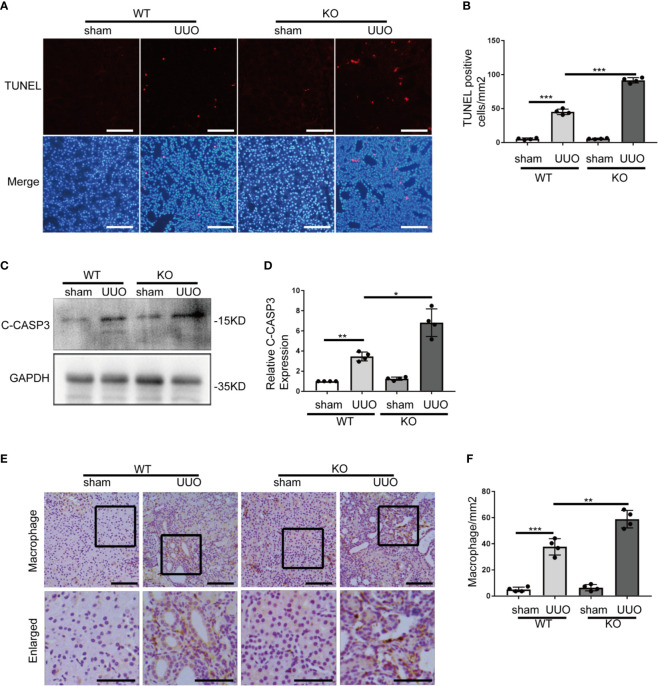
*Park7* deficiency increases tubular cell apoptosis and inflammation in UUO. Animals and their treatment were the same as described in **Figure 2**. **(A)** Representative images of TUNEL analysis of kidney tissues. Bar:100μm. **(B)** Quantification of TUNEL-positive cells in kidney tissues(n=4). **(C)** Representative immunoblots of cleaved/activated caspase3 (C-CASP3) and GAPDH(as a loading control). **(D)** Densitometry of C-CASP3 signals(n=4). The signal of C-CASP3 was normalized to the GAPDH signal of the same samples to determine the ratios. The values are expressed as fold change over sham control in WT mice. **(E)** Representative images of macrophage staining. Bar: 100μm in the top images and 50μm in the bottom enlarged images. **(F)** Quantification of macrophage -positive cells(n=4). Each symbol (circle) represents an individual mouse. Error bars: SEM. *p < 0.05, **p < 0.01, ***p < 0.001.

Tubular cell death and inflammation contribute critically to the development of renal fibrosis regardless of initial etiology (2, 4). The effect of *Park7* deficiency on UUO-induced tubular cell apoptosis was evaluated by TUNEL assay. As shown **Figure 3A**, both WT and *Park7* ko mice had very few TUNEL positive cells in kidney tissues after sham-operation, but they had an obvious increase of TUNEL positive cells following UUO. Moreover, quantification analysis by cell counting revealed that *Park7* ko mice had significantly more TUNEL positive cells than WT mice in response to UUO (**Figure 3B**). Consistent with the results of TUNEL assay, UUO-operated *Park7* ko mice had higher renal levels of activated/cleaved caspase 3 compared to UUO-operated WT mice (**Figures 3C, D**). Immunohistochemical analysis of macrophages in kidney tissue sections was performed to evaluate inflammatory cell infiltration into kidneys. As shown in **Figures 3E, F**, UUO induced infiltration of macrophage into the kidney tissues of both WT and *Park7* ko mice and induced significantly more infiltration of macrophage in *Park7* ko kidneys than in WT kidneys. Collectively, these findings indicate that PARK7 is protective against tubular cell apoptosis and inflammation during renal fibrosis.

### *Park7* Deficiency Leads to Excessive ROS in Renal Tubular Cells Following UUO

**Figure 4:**
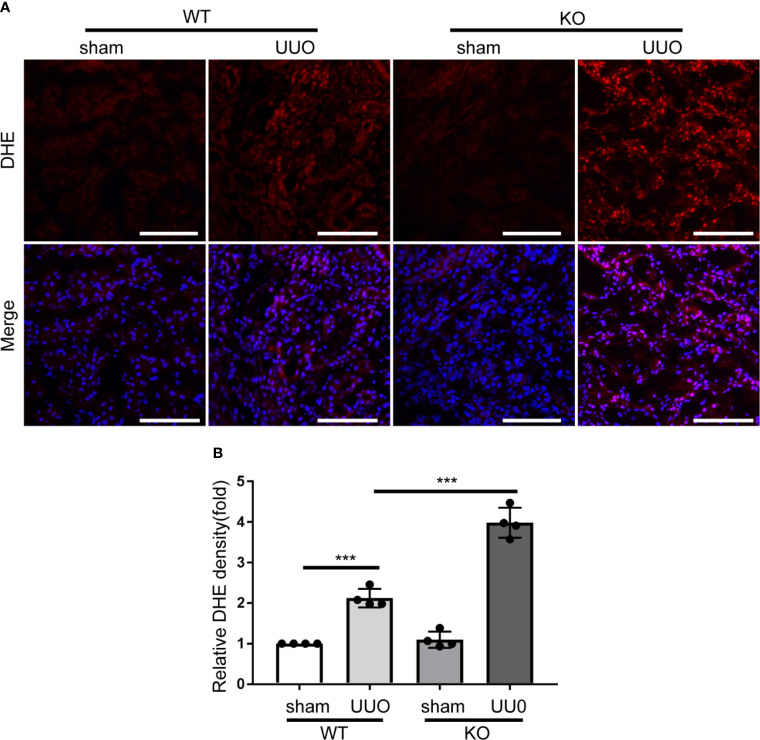
*Park7* deficiency accumulates ROS accumulation in renal tubular cells in UUO. Animals and their treatment were the same as described in **Figure 2**. **(A)** Representative images of dihydroethidium ****(DHE) staining. DHE nuclear staining indicates the presence of reactive oxygen species(ROS). Bar:100μm. **(B)** Quantification of DHE fluorescence intensity (n=4). Each symbol (circle) represents an individual mouse. Error bars: SEM. ***p < 0.001.

PARK7 can act as an antioxidant (29, 30). We thus determined the effect of *Park7* deficiency on the levels of ROS in renal tubules during UUO-induced renal fibrosis by dihydroethidium (DHE) staining. DHE exhibits red fluorescence in the nucleus when oxidized by ROS. WT and *Park7* ko mice showed comparable low DHE signal intensity in renal tubules after sham-operation, and both WT and mutant mice had a dramatic increase of DHE signal intensity after UUO. Importantly, following UUO, *Park7* ko mice exhibited significantly stronger DHE signal intensity in renal tubules compared to WT mice (**Figures 4A, B**). Taken together, these findings suggest that PARK7 suppresses ROS accumulation during renal TIF.

### TGFB1 Treatment Alters PARK7 Expression in BUMPT cells

**Figure 5:**
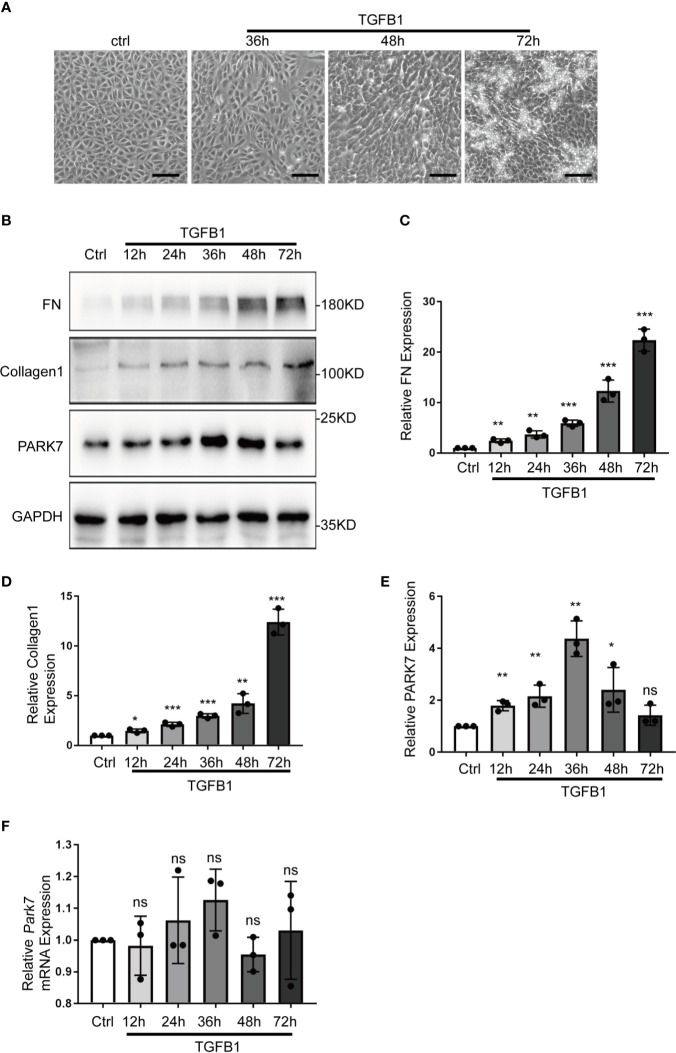
TGFB1 treatment altered PARK7 expression in BUMPT cells. BUMPT cells were incubated with 5 ng/ml TGFB1 for 12h to 72h, and the cells treated without TGFB1 were as control (Ctrl). **(A)** Representative images of contrast microscopy to show cellular morphologic changes. Bar:100μm. **(B)** Representative immunoblot of Fibronectin (FN), Collagen 1, PARK7 and GAPDH (protein loading control) in whole cell lysates. **(C–E)** Densitometry of FN, Collagen 1 and PARK7(n=3). The signal of FN, Collagen 1, or PARK7 was normalized to the GAPDH signal of the same samples to determine the ratios. The values are expressed as fold change over control cells. Each symbol (circle) represents an independent experiment. **(F)** Quantification of Park7 mRNA levels. Error bars: SEM. *p < 0.05, **p < 0.01, ***p < 0.001. ns, not significant.

Transforming growth factor beta 1(TGFB1 or TGF-β1) is a critical profibrotic factor in renal fibrosis. (31) We determined the effect of TGFB1 treatment on PARK7 expression *in vitro*. BUMPT cells formed cobblestone monolayer with intact cell-cell connection under control condition, but, after TGFB1 treatment for 36 hours, the cells exhibited a spindle-shape, fibroblast like morphology(**Figure 5A**). Immunoblot analysis demonstrated that TGFB1 treatment increased the expression of fibrosis maker proteins, such as fibronectin and collagen 1, in a time-dependent manner during the observation period of 72 hours. Remarkably, TGFB1 induced a biphasic change in PARK7 expression. PARK7 increased in a time-dependent manner during 36 hours of TGFB1 treatment, but after that, it started to decrease (**Figures 5B–E**). In addition, We estimated the mRNA level of *Park7* and found that TGFB1 did not change its transcriptional process (**Figure 5F**).

### *Park7* Knockdown Sensitizes BUMPT Cells to TGFB1-Induced Fibrotic Changes, Apoptosis, and ROS Production

**Figure 6:**
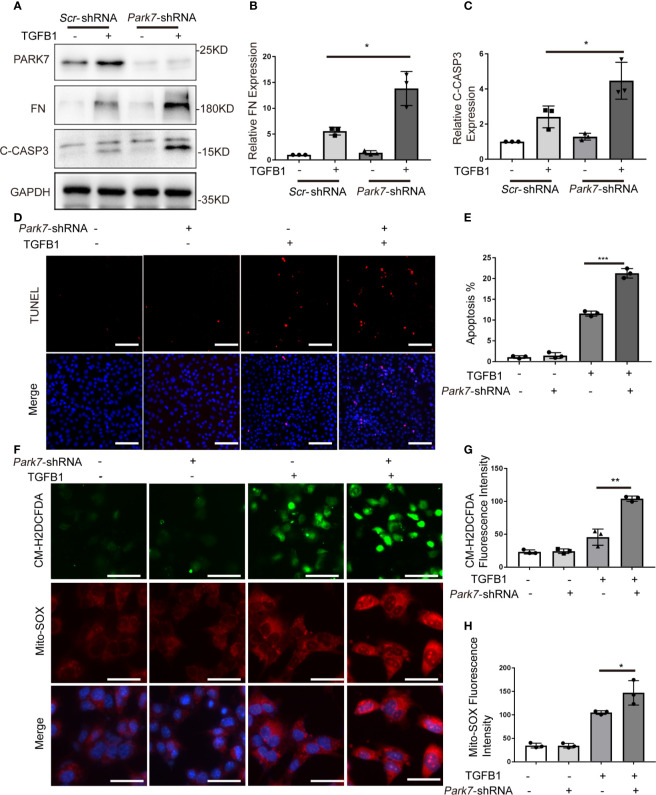
*Park7* knockdown sensitizes BUMPT cells to TGFB1-induced fibrotic changes, apoptosis and ROS production. BUMPT cells were transfected with *Park7-* shRNA or scrambled-shRNA(*Scr*-shRNA), and at 24 hours after transfection, the cells were incubated with/without 5 ng/ml TGFB1 for another 24 hours. **(A)** Representative immunoblot of PARK7, Fibronectin (FN), cleaved/activated caspase 3(C-CASP3) and GAPDH (protein loading control). **(B, C)** Densitometry of FN and C-CASP3(n=3). The signal of FN, or C-CASP3 was normalized to the GAPDH signal of the same samples to determine the ratios. The values are expressed as fold change over control cells without *Park7* shRNA transfection. **(D)** Representative images of TUNEL assay and DAPI staining (X200). Bar:100μm. **(E)** Apoptosis percentage(n=3). **(F)** Representative images of CM-H2DCFDA and Mito-SOX staining. Bar: 100μm in the CM-H2DCFDA image, 50μm in the Mito-SOX and Merge images. CM-H2DCFDA and Mito-SOX staining was performed to evaluate intracellular and mitochondrial ROS, respectively. **(G, H)** Quantification of CM-H2DCFDA and Mito-SOX fluorescence intensity(n=3). Each symbol (circle, diamond and trigon) represents an independent experiment. Error bars: SEM. *p < 0.05, **p < 0.01, ***p < 0.001.

To determine the role of PARK7 in TGFB1-induced changes in renal tubular cells, we knocked down *Park7* with a short hairpin (sh) RNA specifically targeting *Park7*. Immunoblot analysis showed that *Park7*-shRNA significantly reduced PARK7 expression in BUMPT cells (**Figure 6A**). In response to TGFB1 treatment, cells expressing *Park7*-shRNA had higher levels of fibronectin and active/cleaved caspase 3 than the cells transfected with a scrambled-shRNA(*Scr*-shRNA) (**Figures 6A–C**). *Park7* knockdown also increased the number of TUNEL-positive cells following TGFB1 treatment (**Figures 6D, E**). We further analyzed intracellular ROS and mitochondrial ROS by CM-H2DCFDA and Mito-SOX staining, respectively. Regardless of the status of *Park7*, BUMPT cells showed comparable low signal intensity of these ROS indicators under control conditions (**Figures 6F–H**). TGFB1 induced significant ROS, and totably, the *Park7*-knockdown cells had stronger signal intensity of CM-H2DCFDA and Mito-SOX than the cells transfected with scrambled-shRNA. Collectively, these findings suggest that PARK7 may protect against renal fibrosis by reducing ROS and suppressing cell death.

### PARK7 Overexpression Attenuates TGFB1-Induced Profibrotic Changes, Apoptosis, and ROS Production

**Figure 7:**
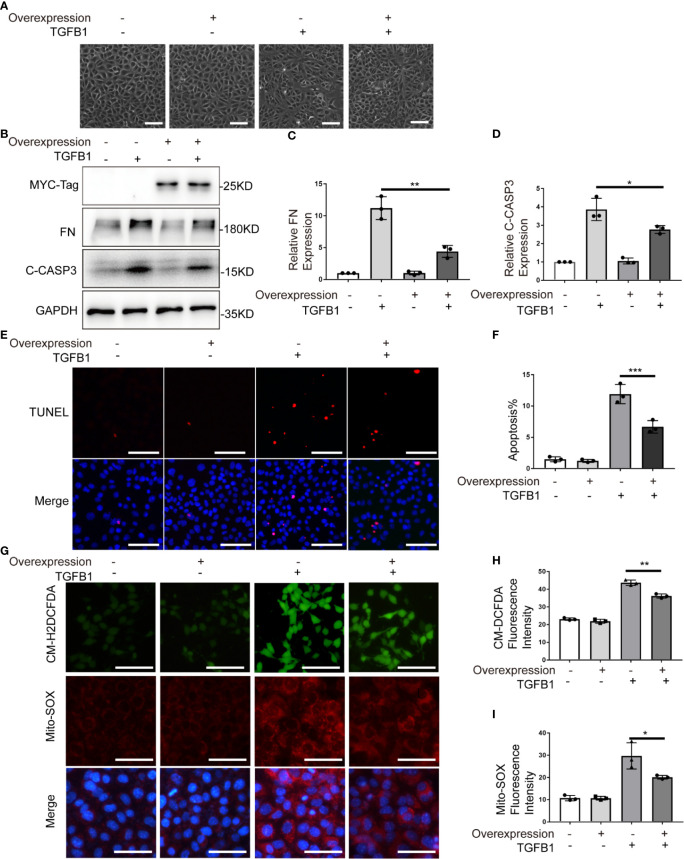
PARK7 overexpression attenuates TGFB1-induced profibrotic changes, apoptosis and ROS production. BUMPT cells were transfected with PARK7 overexpression plasmid or empty vector, and 24 hours after transfection, the cells were cultured in medium with/without 5 ng/ml TGFB1 for another 24 hours. **(A)** Representative images of cell morphology. Bar: 100μm. **(B)** Representative immunoblot of Fibronectin (FN), cleaved/activated caspase 3(C-CASP3), MCY-Tag and GAPDH (protein loading control). **(C, D)** Densitometry of FN and C-CASP3 (n=3). The signal of FN, or C-CASP3 was normalized to the GAPDH signal of the same samples to determine the ratios. The values are expressed as fold change over control cells without *Park7* overexpression. **(E)** Representative images of TUNEL assay and DAPI staining (X200). Bar: 100μm. **(F)** Apoptosis percentage (n=3). Greater than 200 cells in each group were evaluated to determine the percentage of TUNEL-positive cells. **(G)** Representative images of CM-H2DCFDA and Mito-SOX staining. Bar: 100μm in the CM-H2DCFDA image, 50μm in the Mito-SOX and Merge images. CM-H2DCFDA and Mito-SOX staining was performed to evaluate intracellular and mitochondrial ROS, respectively. **(H, I)** Quantification of CM-H2DCFDA and Mito-SOX fluorescence intensity (n=3). Each symbol (circle, diamond and trigon) represents an independent experiment. Error bars: SEM. *p < 0.05, **p < 0.01, ***p < 0.001.

To further verify the role of PARK7, we evaluated the effect of PARK7 overexpression on TGFB1-induced cellular changes in renal tubular cells. To this end, BUMPT cells stably expressing human PARK7 conjugated with a C-terminal c-Myc epitope were generated. As shown in **Figure 7A**, TGFB1 induced a spindle-shape morphology in the cells stablely transfected with empty vectors, which was partially prevented in cells overexpressing PARK7. In addition, BUMPT cells overexpressing PARK7 had lower levels of fibronectin and cleaved caspase 3 compared to control cells (**Figures 7B–D**). In line with the results of cleaved caspase 3, cells overexpression PARK7 had fewer TUNEL-staining positive cells than control cells following TGFB1 treatment(**Figures 7E, F**). In addition, cells overexpressing PARK7 showed lower signal intensity of both CM-H2DCFDA and Mito-SOX staining than control cells in response to TGFB1 treatment(**Figures 7G–I**). Together, these findings provide further evidence that PARK7 protects against apoptosis, ROS production and fibrotic change in renal tubular cells.

### ND-13 Attenuates TGFB1-Induced Fibrosis Changes in BUMPT Cells

**Figure 8:**
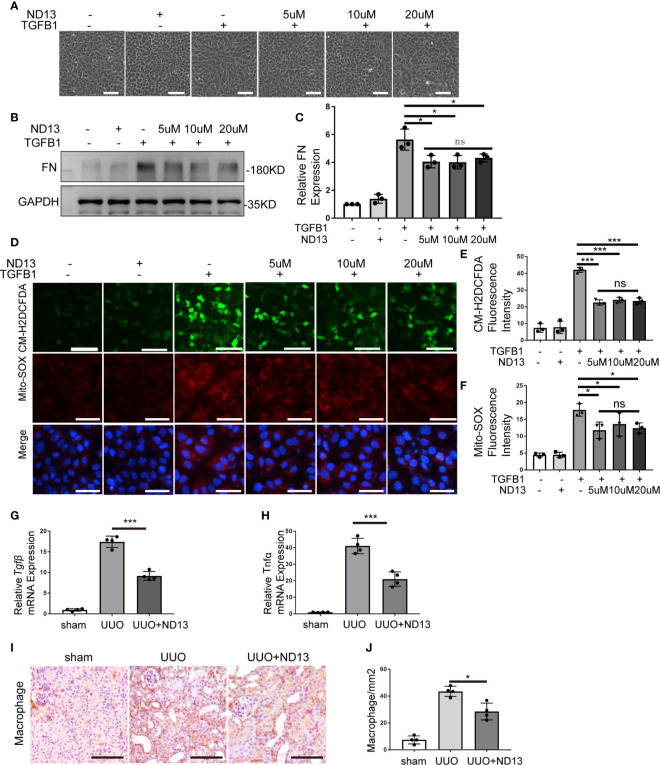
ND-13 attenuates TGFB1-induced fibrosis changes in BUMPT cells. **(A–F)** BUMPT cells were cultured in medium with/without 5ng/ml TGFB1 in the presence/absence of ND13 for 24 hours. **(A)** Representative images of cell morphology. Bar: 100μm. **(B)** Representative immunoblot of Fibronectin (FN), GAPDH (protein loading control). **(C)** Densitometry of FN (n=3). The signal of FN was normalized to the GAPDH signal of the same samples to determine the ratios. The values are expressed as fold change over control cells. **(D)** Representative images of CM-H2DCFDA and Mito-SOX staining. Bar: 100μm in the CM-H2DCFDA image, 50μm in the Mito-SOX and Merge images. CM-H2DCFDA and Mito-SOX staining was performed to evaluate intracellular and mitochondrial ROS, respectively. **(E, F)** Quantification of CM-H2DCFDA and Mito-SOX fluorescence intensity (n=3). **(G–J)** C57BL/6 mice (female, 8weeks old) were subjected to sham operation or UUO surgery. Administration of ND-13 (2 mg/kg of body weight) *via* tail veil injection starting from the day of surgery and daily thereafter. The mice were euthanized at 7 days after surgery, and the UUO-obstructed kidneys were collected. **(G, H)** mRNA levels of transforming growth factor-β (*Tgfβ*) and tumor necrosis factor alpha (*Tnfα*) were measured by quantitative RT-PCR. **(I)** Representative images of macrophage staining. Bar: 100μm. **(J)** Quantification of macrophage -positive cells (n=4). Each symbol (circle, diamond and trigon) represents an independent experiment. Error bars: SEM. *p < 0.05, ***p < 0.001, ns not significant.

ND-13 is a 20-amino acid peptide composed of a 13-amino acids sequence from PARK7 (KGAEEMETVIPVD) and a TAT-derived 7-amino acid sequence (YGRKKRR) at the N-terminus for cellular permeability (32). We first evaluated the effect of ND-13 on the TGFB1-induced cellular changes *in vitro*. To this end, BUMPT cells were exposed to TGFB1 with/without 5~20μM of ND-13 for 24 hours to examine cell morphology and the levels of fibronectin and ROS. As shown in **Figures 8A–F**, ND-13 treatment partially prevented TGFB1-induced morphological changes in BUMPT cell, attenuated TGFB1-induced expression of fibronectin, and also reduced the signal intensity of both CM-H2DCFDA and Mito-SOX staining. We further evaluated the effect of ND-13 treatment on UUO-induced renal fibrosis. Administration of ND-13 (2 mg/kg of body weight) *via* tail veil injection starting from the day of surgery, daily for 7 days partially reduced the renal levels of *Tgfβ* mRNA in UUO mice (**Figure 8G**). ND-13 treatment also significantly reduced the expression of tumor necrosis factor alpha (TNF-α), and also reduced infiltration of macrophages into kidney tissues (**Figures 8H–J**). Taken together, these findings indicate a renoprotective role ND-13 against oxidative stress, inflammation, and renal fibrosis.

### PARK7 Translocates Into Nucleus and Positively Regulates SOD2 Expression During Renal Fibrosis

**Figure 9:**
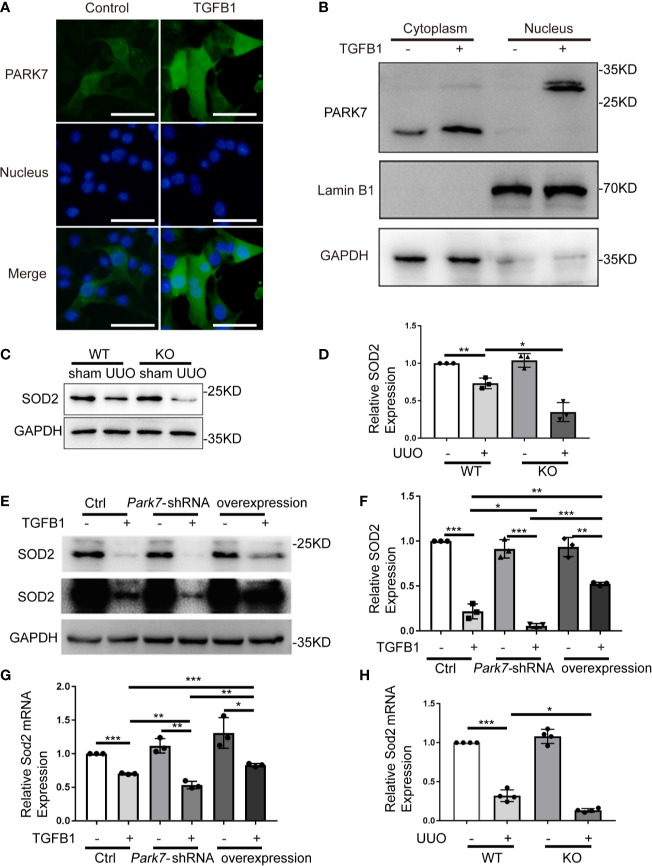
PARK7 translocates into nucleus and positively regulates SOD2 expression during renal fibrosis. **(A, B)** BUMPT cells were cultured in medium with/without 5 ng/ml TGFB1 for 24 hours. **(A)** Representative images of immunofluorescence staining of PARK7 in BUMPT cells. Bar:50μm. **(B)** Representative immunoblot of PARK7, LaminB1 (nuclear marker), and GAPDH (cytosolic marker) in cytosolic and nuclear fractions. **(C, D)** Animals and their treatment were the same as described in **Figure 2**. **(C)** Representative immunoblot of SOD2 and GAPDH. **(D)** Densitometry of SOD2 signals (n=4). **(E, F)** BUMPT cells were transfected with *Park7* siRNA or PARK7 overexpression plasmid. 24 hours after transfection, the cells were cultured in medium with or without 5 ng/ml TGFB1 for another 24 hours. **(E)** Representative immunoblot of SOD2 and GAPDH. **(F)** Densitometry of SOD2 signals (n=3). The signal of SOD2 was normalized to the GAPDH signal of the same samples to determine the ratios. The values are expressed as fold change over control cells. **(G, H)** *Sod2* mRNA expression. Each symbol (circle, diamond and trigon) represents an independent experiment or an individual mouse. Error bars: SEM. *p < 0.05, **p < 0.01, ***p < 0.001.

In UUO kidneys, PARK7 showed nuclear accumulation in intact renal tubules (**Figure 1A**). We thus examined the nuclear levels of PARK7 in BUMPT cell with or without TGFB1 treatment. Immunofluorescence staining showed that in cells without TGFB1 treatment, PARK7 existed mainly in cytosol, and to less extent in the nucleus (**Figure 9A**). Immunoblotting of PARK7 in the nuclear and cytoplasmic fractions verified that PARK7 localized predominantly in the cytosol in control cells and showed a dramatical increase in both cytosolic and nuclear fractions following TGFB1 treatment (**Figure 9B**). Emerging evidence indicates that nuclear PARK7 may regulate gene transcription by acting as a transcriptional cofactor (33). Because PARK7 regulated ROS production in mitochondria (**Figures 6F** and **7G**), we tested the possible regulation of SOD2 (mitochondrial superoxide dismutase 2, also known as manganese-dependent superoxide dismutase or MnSOD) by PARK7. As shown in **Figures 9C, D**, both WT and *Park7* ko mice showed high levels of SOD2 in kidney tissues, which decreased during UUO. Notably, the SOD2 decrease was obviously more in *Park7* ko mice than in WT mice. Consistently, TGFB1 treatment reduced SOD2 expression in both *Park7* knockdown cells and control cells, but the reduction was significantly higher in *Park7* knockdown cells. Moreover, *PARK7* overexpression could partially but significantly restore SOD2 levels in TGFB1-treated cells (**Figures 9E, F**). Furthermore, we analyzed *Sod2* mRNA, which showed the regulation by PARK7 both *in vitro* and *in vivo* (**Figures 9G, H**). Collectively, these findings suggest that PARK7 may protect against chronic kidney injury and renal fibrosis by inducing SOD2 and reducing ROS and oxidative stress.

## Discussion

In the present study, we have provided substantial evidence supporting a renoprotective role of renal tubular cell PARK7 in renal fibrosis. First, we showed that PARK7 expression was altered in renal tubules in UUO mice and in BUMPT cells exposed to TGFB1. Second, we demonstrated that *Park7* deficiency aggravated UUO-induced renal fibrosis in mice. *Park7* knockdown in BUMPT cells exacerbated TGFB1-induced fibrotic changes, while *PARK7* overexpression attenuated the fibrotic changes. Third, we provided further evidence that *Park7* deficiency or knockdown aggravated ROS accumulation, tubular cell apoptosis, and inflammation in renal fibrosis models. Mechanistically, we showed that during renal fibrosis, PARK7 accumulated in the nucleus in renal tubular cells, and positively regulated expression of the antioxidant enzyme SOD2. Together, these findings suggest a protective function of PARK7 in chronic kidney injury and renal fibrosis, and accordingly, enhancement of PARK7 expression and/or activity may represent a promising therapeutic approach.

Recent studies have suggested a role of PARK7 in the development of kidney diseases. For instance, Leeds et al. demonstrated that activation of PARK7 was renoprotective in lipopolysaccharides- or cecal ligation and puncture-induced septic AKI in mice (34). Shen et al. showed that increased expression of renal tubular PARK7 might represent a renoprotective response in a rat model of high glucose-induced diabetic kidney diseases (35). However, the pathophysiological role and mechanism of PARK7 in the pathogenesis of renal fibrosis remain largely unclear. Our present study has provided evidence supporting a beneficial role of renal tubular PARK7 in renal fibrosis. In *vitro*, TGFB1-induced fibrotic changes in BUMPT cells, including cell morphological changes and increases of extracellular matrix proteins were enhanced by *PARK7* knockdown (**Figures 6A, B**), but attenuated by *PARK7* overexpression (**Figures 7A–C**). In *vivo*, *Park7* deficiency aggravated UUO-induced renal fibrosis, as manifested by more severe tubular atrophy and kidney deposition of extracellular matrix proteins in *Park7* ko mice than in WT mice following UUO (**Figure 2**). Consistently, the latest work by Miguel et al. demonstrated the inhibitory effect of ND-13 (PARK7 peptide) on UUO-induced renal fibrosis (36). Collectively, these studies indicate that renal tubular cell PARK7 has an anti-fibrotic, protective function in kidney diseases.

PARK7 expression appears to be differently altered according to diseases conditions, and/or cell types (37, 38). For instance, PARK7 is highly expressed in lipopolysaccharides-induced acute lung injury (39), while decreased in heavy smoking-induced lung disease (40). Compared to control animals, altered PARK7 levels in kidney tissues have also demonstrated in experimental animal of kidney diseases. An increase of PARK7 levels in kidney tissues was detected in in mouse models of septic AKI, and a ischemia-reperfusion-induced rat model in diabetic kidney injury (34, 41). Eltoweissy et al. showed a disease grade dependent increase of PARK7 levels in the kidneys of a mouse model of COL4A3-deficiency-induced renal fibrosis (42). Consistently, Eltoweissy et al. also revealed that treatment of renal epithelial cells with the profibrogenic agonist ANG II or PDGF resulted in a significant up-regulation of PARK7 expression parallel to an increase in the expression of fibrosis markers (42). In a sharp contrast, in the present study, immunoblot analysis revealed that UUO mice had a dramatic reduction of PARK7 levels in kidney tissues compared to sham-operated mice (**Figures 1B, E**). Immunohistochemical staining further demonstrated that PARK7 decreased in atrophic renal tubules in UUO mice, but it increased in the intact renal tubules (**Figure 1A**). Further evaluation of *Park7* mRNA levels revealed no differences between UUO- and sham-operated mice (**Figure 1F**). In BUMPT cells, we showed an initial increase of PARK7 followed by a decrease during TGFB1 treatment (**Figures 5B, E**), but TGFB1 had no effect on the levels of *Park7* mRNA (**Figure 5F**). These results suggest an increased degradation of PARK7 in UUO, especially in those atrophic tubules. The different alteration of kidney expression of PARK7 between the present study and Eltoweissy et al. study may be attributed to the differences in the experimental models. Recent evidences have implicated that PARK7 oxidation facilitates its degradation (43, 44). In the present study, accumulation of ROS occurred in atrophic renal tubules in UUO mice as evaluated by DHE staining analysis (**Figure 4**). However, PARK7 oxidation and its contribution to PARK7 degradation during renal fibrosis remain to be determined. In addition, RNA modifications have been shown to affect the outcome of gene expression by modulating RNA processing, localization, translation, and eventual decay (45, 46). Whether modification in *Park7* mRNA contributes to the PARK7 reduction without changing the *Park7* mRNA levels in renal tubules of UUO mice remain to be explored.

PARK7 has multiple functions including antioxidant, transcriptional co-activator, molecular chaperone, deglycase, and/or regulator of signaling pathways (15). Among them, the antioxidant capacity has been demonstrated as an important cytoprotective mechanism in various disease conditions, such as PARK7 protection of rat retinal pericytes against high glucose-induced oxidative stress (47). Accumulation of ROS in renal tubules is a comment feature of chronic kidney disease, and contributes critically to the development to both AKI and CKD (48). High levels of ROS cause oxidative damage to cellular components, which may increase the tendency of cell injury and death (49). ROS are also an activator of inflammation (50). In the present study, we provided evidence that PARK7 has an important role in scavenging ROS and thereby reduces cell death and inflammation in kidneys during renal fibrosis. In *vivo*, *Park7* deficiency led to the excessive accumulation of ROS (**Figure 4**), increased renal tubular cell apoptosis and inflammation in UUO mice (**Figure 3**). Consistently, *Park7* knockdown increased the levels of ROS and apoptosis (**Figure 6**), while *PARK7* overexpression showed opposite effects (**Figure 7**). Thus, PARK7 plays an important role in scavenging ROS in renal tubules during renal fibrosis.

PARK7 can act as a transcription cofactor, a molecular chaperon, and can also modulate signaling pathway involving stress response, and also regulate protein degradation, all of which may contribute to its regulation of protein expression (51). For instance, PARK7 has been shown to disrupt KEAP1 interaction with transcriptional factors NRF2, leading to nuclear translocation of NRF2 to transactivate various antioxidative stress-related genes (52). PARK7 has also been demonstrated to regulate the 20S proteasome and thus protein degradation (53). In our experimental setting, nuclear accumulation of PARK7 was detected in the intact renal tubule of UUO mice and BUMPT cells exposed to TGFB1 (**Figures 1A** and **9A, B**). In addition, PARK7 seemed to positively regulate the protein levels of enzymatic antioxidant SOD2 (**Figures 9C–H**). However, the precise mechanism responsible for the regulatory role of PARK7 on SOD2 awaits future investigation.

ND-13 is PARK7-based peptide that has been shown be cytoprotective under stressful conditions, such as Cerebral reperfusion injury (32). Miguel et al. showed that UUO mice received ND-13 (3 mg/kg of body weight) *via* subcutaneous injection starting from the day of surgery, daily for 14 days showed reduced expression of fibrotic and inflammatory markers compared to UUO mice received vehicle, suggesting a beneficial effect of ND-13 in renal fibrosis (36). In the present study, administration of ND-13 (2 mg/kg of body weight) *via* tail veil injection starting from the day after surgery, daily for 7 days reduced the renal levels of *Tgfβ* mRNA, *Tnfα* mRNA and infiltration of macrophages into kidney tissues (**Figures 8G–J**). In addition, we showed that ND-13 treatment reduced TGFB1-induced fibrotic changes in BUMPT cells (**Figures 8A–C**). Thus, ND-13 may represent a potential drug for renal fibrosis.

In summary, this study provide evidence that PARK7 in renal tubular cells is renoprotective during renal fibrosis through suppressing ROS accumulation, cell apoptosis and inflammation. Thus, prevention of ROS accumulation *via* enhancing intracellular PARK7 levels and/or activity represents potential therapeutic strategies for renal fibrosis.

## Data Availability Statement

The original contributions presented in the study are included in the article/supplementary material. Further inquiries can be directed to the corresponding authors.

## Ethics Statement

The animal study was reviewed and approved by The Animal Ethics Committee of the Second Xiangya Hospital of Central South University.

## Author Contributions

ZD, CT and LY conceived the study. LY performed the experiments. LY, HL, ZL, WW, JC, CT and ZD analyzed the results and approved the publication. LY drafted the manuscript. ZD and CT revised the paper. All authors contributed to the article and approved the submitted version.

## Funding

This study was supported partly by the Natural Science Foundation of China (Grant Number 81870474), the National Key R&D Program of China (2018YFC1312700), and the Natural Science Foundation of Hunan Province (2019JJ40415).

## Conflict of Interest

The authors declare that the research was conducted in the absence of any commercial or financial relationships that could be construed as a potential conflict of interest.

## References

[1] Liu Y. Cellular and Molecular Mechanisms of Renal Fibrosis. Nat Rev Nephrol (2011) 7(12):684–96. 10.1038/nrneph.2011.149 PMC4520424 22009250

[2] Venkatachalam MA Weinberg JM Kriz W Bidani AK. Failed Tubule Recovery, AKI-CKD Transition, and Kidney Disease Progression. J Am Soc Nephrol (2015) 26(8):1765–76. 10.1681/asn.2015010006 PMC4520181 25810494

[3] Humphreys BD. Mechanisms of Renal Fibrosis. Annu Rev Physiol (2018) 80:309–26. 10.1146/annurev-physiol-022516-034227 29068765

[4] Liu BC Tang TT Lv LL Lan HY. Renal Tubule Injury: A Driving Force Toward Chronic Kidney Disease. Kidney Int (2018) 93(3):568–79. 10.1016/j.kint.2017.09.033 29361307

[5] Yang L Besschetnova TY Brooks CR Shah JV Bonventre JV. Epithelial Cell Cycle Arrest in G2/M Mediates Kidney Fibrosis After Injury. Nat Med (2010) 16(5):535–43. 10.1038/nm.2144. 531p following 143. PMC3928013 20436483

[6] Shu S Zhu J Liu Z Tang C Cai J Dong Z. Endoplasmic Reticulum Stress is Activated in Post-Ischemic Kidneys to Promote Chronic Kidney Disease. EBioMedicine (2018) 37:269–80. 10.1016/j.ebiom.2018.10.006 PMC6286638 30314894

[7] Tang C Cai J Yin XM Weinberg JM Venkatachalam MA Dong Z. Mitochondrial Quality Control in Kidney Injury and Repair. Nat Rev Nephrol (2020a) 17(5):299–318. 10.1038/s41581-020-00369-0 33235391 PMC8958893

[8] Tang C Livingston MJ Liu Z Dong Z. Autophagy in Kidney Homeostasis and Disease. Nat Rev Nephrol (2020b) 16(9):489–508. 10.1038/s41581-020-0309-2 32704047 PMC7868042

[9] Basile DP Leonard EC Beal AG Schleuter D Friedrich J. Persistent Oxidative Stress Following Renal Ischemia-Reperfusion Injury Increases ANG II Hemodynamic and Fibrotic Activity. Am J Physiol Renal Physiol (2012) 302(11):F1494–1502. 10.1152/ajprenal.00691.2011 PMC3378170 22442209

[10] Finkel T. Signal Transduction by Reactive Oxygen Species. J Cell Biol (2011) 194(1):7–15. 10.1083/jcb.201102095 21746850 PMC3135394

[11] Nagakubo D Taira T Kitaura H Ikeda M Tamai K Iguchi-Ariga SM. Dj-1, a Novel Oncogene Which Transforms Mouse NIH3T3 Cells in Cooperation With Ras. Biochem Biophys Res Commun (1997) 231(2):509–13. 10.1006/bbrc.1997.6132 9070310

[12] Honbou K Suzuki NN Horiuchi M Niki T Taira T Ariga H. The Crystal Structure of DJ-1, a Protein Related to Male Fertility and Parkinson’s Disease. J Biol Chem (2003) 278(33):31380–4. 10.1074/jbc.M305878200 12796482

[13] Zhou B Lei S Xue R Leng Y Xia Z Xia ZY. DJ-1 Overexpression Restores Ischaemic Post-Conditioning-Mediated Cardioprotection in Diabetic Rats: Role of Autophagy. Clin Sci (Lond) (2017) 131(11):1161–78. 10.1042/cs20170052 28404768

[14] Zhang L Wang J Wang J Yang B He Q Weng Q. Role of DJ-1 in Immune and Inflammatory Diseases. Front Immunol (2020) 11:994. 10.3389/fimmu.2020.00994 32612601 PMC7308417

[15] Ariga H Takahashi-Niki K Kato I Maita H Niki T Iguchi-Ariga SM. Neuroprotective Function of DJ-1 in Parkinson’s Disease. Oxid Med Cell Longev (2013) 2013:683920. 10.1155/2013/683920 23766857 PMC3671546

[16] Cuevas S Yang Y Konkalmatt P Asico LD Feranil J Jones J. Role of Nuclear Factor Erythroid 2-Related Factor 2 in the Oxidative Stress-Dependent Hypertension Associated With the Depletion of DJ-1. Hypertension (2015) 65(6):1251–7. 10.1161/hypertensionaha.114.04525 PMC4433423 25895590

[17] Sun Q Shen ZY Duan WN Meng QT Xia ZY. Mechanism of Myocardial Ischemia/Reperfusion-Induced Acute Kidney Injury Through DJ-1/Nrf2 Pathway in Diabetic Rats. Exp Ther Med (2017) 14(5):4201–7. 10.3892/etm.2017.5095 PMC5658721 29104636

[18] Qu XH Zhang K. MiR-122 Regulates Cell Apoptosis and ROS by Targeting DJ-1 in Renal Ischemic Reperfusion Injury Rat Models. Eur Rev Med Pharmacol Sci (2018) 22(24):8830–8. 10.26355/eurrev_201812_16651 30575925

[19] Yin J Xu R Wei J Zhang S. The Protective Effect of Glutaredoxin 1/DJ-1/HSP70 Signaling in Renal Tubular Epithelial Cells Injury Induced by Ischemia. Life Sci (2019) 223:88–94. 10.1016/j.lfs.2019.03.015 30858124

[20] Tsai BC Hsieh DJ Lin WT Tamilselvi S Day CH Ho TJ. Functional Potato Bioactive Peptide Intensifies Nrf2-dependent Antioxidant Defense Against Renal Damage in Hypertensive Rats. Food Res Int (2020) 129:108862. 10.1016/j.foodres.2019.108862 32036911

[21] Sinha D Wang Z Price VR Schwartz JH Lieberthal W. Chemical Anoxia of Tubular Cells Induces Activation of c-Src and its Translocation to the Zonula Adherens. Am J Physiol Renal Physiol (2003) 284(3):F488–97. 10.1152/ajprenal.00172.2002 12419774

[22] Livingston MJ Ding HF Huang S Hill JA Yin XM Dong Z. Persistent Activation of Autophagy in Kidney Tubular Cells Promotes Renal Interstitial Fibrosis During Unilateral Ureteral Obstruction. Autophagy (2016) 12(6):976–98. 10.1080/15548627.2016.1166317 PMC4922446 27123926

[23] Cai J Liu Z Huang X Shu S Hu X Zheng M. The Deacetylase Sirtuin 6 Protects Against Kidney Fibrosis by Epigenetically Blocking β-Catenin Target Gene Expression. Kidney Int (2020) 97(1):106–18. 10.1016/j.kint.2019.08.028 31787254

[24] Zheng M Cai J Liu Z Shu S Wang Y Tang C. Nicotinamide Reduces Renal Interstitial Fibrosis by Suppressing Tubular Injury and Inflammation. J Cell Mol Med (2019) 23(6):3995–4004. 10.1111/jcmm.14285 30993884 PMC6533567

[25] Tang C Han H Yan M Zhu S Liu J Liu Z. Pink1-PRKN/PARK2 Pathway of Mitophagy is Activated to Protect Against Renal Ischemia-Reperfusion Injury. Autophagy (2018) 14(5):880–97. 10.1080/15548627.2017.1405880 PMC6070003 29172924

[26] Tang C Han H Liu Z Liu Y Yin L Cai J. Activation of BNIP3-mediated Mitophagy Protects Against Renal Ischemia-Reperfusion Injury. Cell Death Dis (2019) 10(9):677. 10.1038/s41419-019-1899-0 31515472 PMC6742651

[27] Wang Y Tang C Cai J Chen G Zhang D Zhang Z. PINK1/Parkin-Mediated Mitophagy is Activated in Cisplatin Nephrotoxicity to Protect Against Kidney Injury. Cell Death Dis (2018) 9(11):1113. 10.1038/s41419-018-1152-2 30385753 PMC6212494

[28] Fu Y Cai J Li F Liu Z Shu S Wang Y. Chronic Effects of Repeated Low-Dose Cisplatin Treatment in Mouse Kidneys and Renal Tubular Cells. Am J Physiol Renal Physiol (2019) 317(6):F1582–92. 10.1152/ajprenal.00385.2019 31532246

[29] Yanagida T Tsushima J Kitamura Y Yanagisawa D Takata K Shibaike T. Oxidative Stress Induction of DJ-1 Protein in Reactive Astrocytes Scavenges Free Radicals and Reduces Cell Injury. Oxid Med Cell Longev (2009) 2(1):36–42. 10.4161/oxim.2.1.7985 20046643 PMC2763229

[30] Eltoweissy M Müller GA Bibi A Nguye PV Dihazi GH Müller CA. Proteomics Analysis Identifies PARK7 as an Important Player for Renal Cell Resistance and Survival Under Oxidative Stress. Mol Biosyst (2011) 7(4):1277–88. 10.1039/c0mb00116c 21308111

[31] Meng XM Nikolic-Paterson DJ Lan HY. TGF-Beta: The Master Regulator of Fibrosis. Nat Rev Nephrol (2016) 12(6):325–38. 10.1038/nrneph.2016.48 27108839

[32] Molcho L Ben-Zur T Barhum Y Offen D. DJ-1 Based Peptide, ND-13, Promote Functional Recovery in Mouse Model of Focal Ischemic Injury. PloS One (2018) 13(2):e0192954. 10.1371/journal.pone.0192954 29489843 PMC5831040

[33] Lu L Zhao S Gao G Sun X Zhao H Yang H. Dj-1/Park7, But Not Its L166p Mutant Linked to Autosomal Recessive Parkinsonism, Modulates the Transcriptional Activity of the Orphan Nuclear Receptor Nurr1 In Vitro and In Vivo. Mol Neurobiol (2016) 53(10):7363–74. 10.1007/s12035-016-9772-y 26873851

[34] Leeds J Scindia Y Loi V Wlazlo E Ghias E Cechova S. Protective Role of DJ-1 in Endotoxin-Induced Acute Kidney Injury. Am J Physiol Renal Physiol (2020) 319(4):F654–f663. 10.1152/ajprenal.00064.2020 32715759 PMC7642890

[35] Shen ZY Sun Q Xia ZY Meng QT Lei SQ Zhao B. Overexpression of DJ-1 Reduces Oxidative Stress and Attenuates Hypoxia/Reoxygenation Injury in NRK-52E Cells Exposed to High Glucose. Int J Mol Med (2016) 38(3):729–36. 10.3892/ijmm.2016.2680 PMC4990284 27430285

[36] De Miguel C Kraus AC Saludes MA Konkalmatt P Ruiz Domínguez A Asico LD. Nd-13, a DJ-1-Derived Peptide, Attenuates the Renal Expression of Fibrotic and Inflammatory Markers Associated With Unilateral Ureter Obstruction. Int J Mol Sci (2020) 21(19):7048. 10.3390/ijms21197048 PMC7582723 32987947

[37] Neumann M Müller V Görner K Kretzschmar HA Haass C Kahle PJ. Pathological Properties of the Parkinson’s Disease-Associated Protein DJ-1 in Alpha-Synucleinopathies and Tauopathies: Relevance for Multiple System Atrophy and Pick’s Disease. Acta Neuropathol (2004) 107(6):489–96. 10.1007/s00401-004-0834-2 14991385

[38] Abd El Atti RM Abou Gabal HH Osman WM Saad AS. Insights Into the Prognostic Value of DJ-1 and MIB-1 in Astrocytic Tumors. Diagn Pathol (2013) 8:126. 10.1186/1746-1596-8-126 23902708 PMC3765979

[39] Amatullah H Maron-Gutierrez T Shan Y Gupta S Tsoporis JN Varkouhi AK. Protective Function of DJ-1/PARK7 in Lipopolysaccharide and Ventilator-Induced Acute Lung Injury. Redox Biol (2021) 38:101796. 10.1016/j.redox.2020.101796 33246293 PMC7695876

[40] Bahmed K Messier EM Zhou W Tuder RM Freed CR Chu HW. Dj-1 Modulates Nuclear Erythroid 2-Related Factor-2-Mediated Protection in Human Primary Alveolar Type II Cells in Smokers. Am J Respir Cell Mol Biol (2016) 55(3):439–49. 10.1165/rcmb.2015-0304OC PMC5023027 27093578

[41] Cheng Z Qian S Qingtao M Zhongyuan X Yeda X. Effects of ATRA on Diabetic Rats With Renal Ischemia-Reperfusion Injury. Acta Cir Bras (2020) 35(1):e202000106. 10.1590/s0102-865020200010000006 32236320 PMC7106780

[42] Eltoweissy M Dihazi GH Müller GA Asif AR Dihazi H. Protein DJ-1 and its Anti-Oxidative Stress Function Play an Important Role in Renal Cell Mediated Response to Profibrotic Agents. Mol Biosyst (2016) 12(6):1842–59. 10.1039/c5mb00887e 27109140

[43] Meulener MC Xu K Thomson L Ischiropoulos H Bonini NM. Mutational Analysis of DJ-1 in Drosophila Implicates Functional Inactivation by Oxidative Damage and Aging. Proc Natl Acad Sci USA (2006) 103(33):12517–22. 10.1073/pnas.0601891103 PMC1533799 16894167

[44] Ooe H Maita C Maita H Iguchi-Ariga SM Ariga H. Specific Cleavage of DJ-1 Under an Oxidative Condition. Neurosci Lett (2006) 406(3):165–8. 10.1016/j.neulet.2006.06.067 16935423

[45] Meyer KD Jaffrey SR. The Dynamic Epitranscriptome: N6-methyladenosine and Gene Expression Control. Nat Rev Mol Cell Biol (2014) 15(5):313–26. 10.1038/nrm3785 PMC4393108 24713629

[46] Zaccara S Ries RJ Jaffrey SR. Reading, Writing and Erasing mRNA Methylation. Nat Rev Mol Cell Biol (2019) 20(10):608–24. 10.1038/s41580-019-0168-5 31520073

[47] Wang W Zhao H Chen B. DJ-1 Protects Retinal Pericytes Against High Glucose-Induced Oxidative Stress Through the Nrf2 Signaling Pathway. Sci Rep (2020) 10(1):2477. 10.1038/s41598-020-59408-2 32051471 PMC7016111

[48] Podkowińska A Formanowicz D. Chronic Kidney Disease as Oxidative Stress- and Inflammatory-Mediated Cardiovascular Disease. Antioxidants (Basel) (2020) 9(8):752. 10.3390/antiox9080752 PMC7463588 32823917

[49] Circu ML Aw TY. Reactive Oxygen Species, Cellular Redox Systems, and Apoptosis. Free Radic Biol Med (2010) 48(6):749–62. 10.1016/j.freeradbiomed.2009.12.022 PMC2823977 20045723

[50] Morgan MJ Liu ZG. Crosstalk of Reactive Oxygen Species and NF-κb Signaling. Cell Res (2011) 21(1):103–15. 10.1038/cr.2010.178 PMC3193400 21187859

[51] Biosa A Sandrelli F Beltramini M Greggio E Bubacco L Bisaglia M. Recent Findings on the Physiological Function of DJ-1: Beyond Parkinson’s Disease. Neurobiol Dis (2017) 108:65–72. 10.1016/j.nbd.2017.08.005 28823929

[52] Clements CM McNally RS Conti BJ Mak TW Ting JP. Dj-1, a Cancer- and Parkinson’s Disease-Associated Protein, Stabilizes the Antioxidant Transcriptional Master Regulator Nrf2. Proc Natl Acad Sci USA (2006) 103(41):15091–6. 10.1073/pnas.0607260103 PMC1586179 17015834

[53] Moscovitz O Ben-Nissan G Fainer I Pollack D Mizrachi L Sharon M. The Parkinson’s-Associated Protein DJ-1 Regulates the 20S Proteasome. Nat Commun (2015) 6:6609. 10.1038/ncomms7609 25833141

